# Complications of calcific tendinitis of the shoulder: a concise review

**DOI:** 10.1007/s10195-015-0339-x

**Published:** 2015-02-20

**Authors:** Giovanni Merolla, Mahendar G. Bhat, Paolo Paladini, Giuseppe Porcellini

**Affiliations:** 1 Unit of Shoulder and Elbow Surgery, D. Cervesi Hospital, Cattolica, AUSL della Romagna Ambito Territoriale di Rimini, Rimini, Italy; 2 Biomechanics Laboratory “Marco Simoncelli”, D. Cervesi Hospital, Cattolica, AUSL della Romagna Ambito Territoriale di Rimini, Rimini, Italy

**Keywords:** Calcific tendinitis, Shoulder, Rotator cuff, Complications

## Abstract

Calcific tendinitis (CT) of the rotator cuff (RC) muscles in the shoulder is a disorder which remains asymptomatic in a majority of patients. Once manifested, it can present in different ways which can have negative effects both socially and professionally for the patient. The treatment modalities can be either conservative or surgical. There is poor literature evidence on the complications of this condition with little consensus on the treatment of choice. In this review, the literature was extensively searched in order to study and compile together the complications of CT of the shoulder and present it in a clear form to ease the understanding for all the professionals involved in the management of this disorder. Essentially there are five major complications of CT: pain, adhesive capsulitis, RC tears, greater tuberosity osteolysis and ossifying tendinitis. All the above complications have been explained right from their origin to the control measures required for the relief of the patient.

Level of evidence 5.

## Introduction

Calcifying tendinitis (CT) of the shoulder is a frequently occurring painful disorder characterized by the presence of calcified deposits in the tendons of the rotator cuff (RC) mainly affecting the supraspinatus tendon but occasionally is seen in the infraspinatus and subscapularis [1–5].

**Fig. 1:**
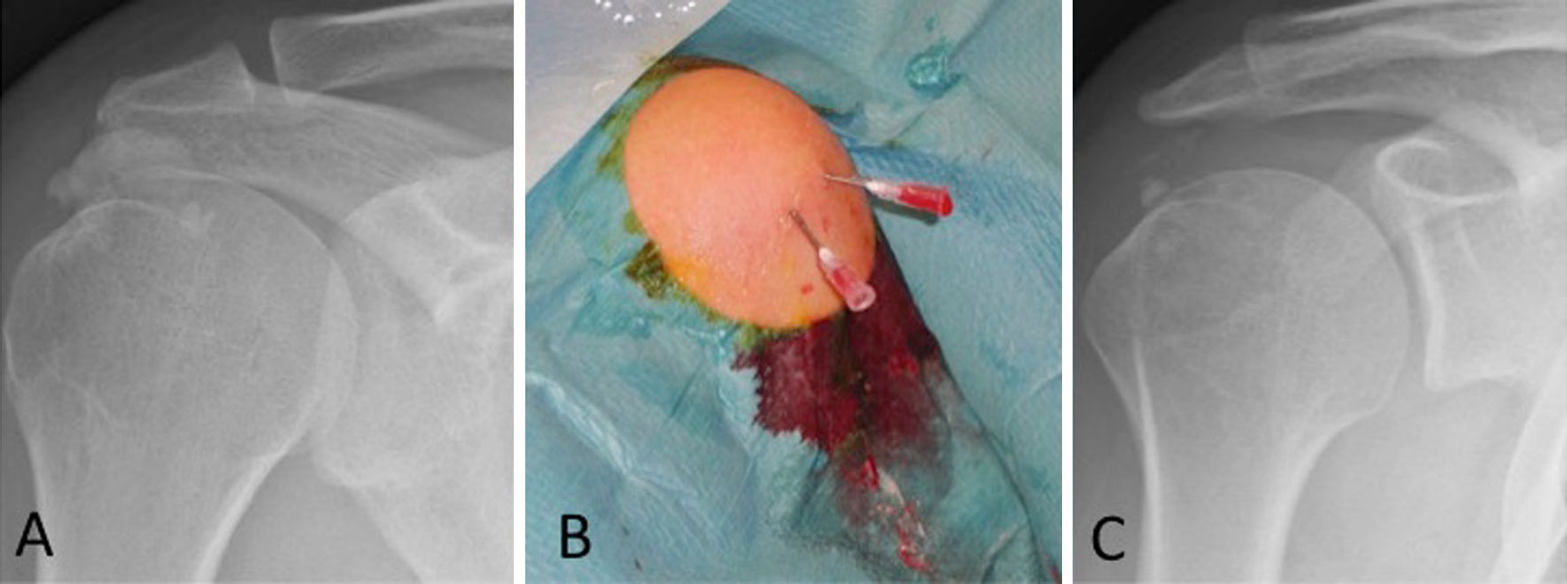
**a** AP view radiograph shows a big calcium deposit (>1 cm) of the supraspinatus (SS) tendon in a case with acute phase, **b** image of the same case who underwent ultrasound guided needling and bursal lavage of the subacromial space with leakage of copious amounts of semisolid calcium deposits, **c** X-ray performed after 2 months from bursal lavage showed almost complete resorption of the calcium deposit

The prevalence has been reported to be 2.7 percent in asymptomatic individuals, more common in females between the 4th and 6th decades of life and in sedentary workers [6, 7]. Two speculative hypotheses have been introduced to explain the etiology of CT [8]. The first one was proposed by Codman as an initial degeneration within the tendon fibers which is followed by calcification [9]. Moseley expanded on this further by defining a ‘‘critical zone’’ in the tendon-bone insertion area [10].The second one was proposed by Uhthoff who considered CT as a reactive calcification within a healthy tendon [11]. CT is a disabling clinical condition that in the acute phase induces severe pain and limitation of shoulder function. Although most cases of CT elapse almost asymptomatically, it is not uncommon that some of them present in an emergency or with frequent outpatient office visits due to the ineffectiveness of the various conservative treatment modalities. CT heals either spontaneously or by conservative methods such as nonsteroidal anti-inflammatory drugs (NSAIDs), physiotherapy, subacromial injections, bursal lavage and extracorporeal shock-wave therapy (ESWT) (Fig. 1a–c) [3, 12–21]. In cases resistant to non operative measures, surgical removal of the calcium deposits is recommended [11, 22–25].

To our knowledge no review articles have been elaborated on the complications of CT. Hence, in this paper a literature review has been done on the various complications or sequelae of the CT of the shoulder preceded by a brief overview on its histopathology, classification and diagnostic imaging.

## Histopathology and classification

The evolution of CT essentially passes through 3 distinct stages: pre-calcific, calcifying and post-calcific [26]. In the pre-calcific stage, numerous factors stimulate a metaplastic change of the tenocytes into chondrocytes. This is followed by the calcific stage which is subdivided into three phases—formation, resting and resorption—characterized by deposition of amorphous calcium phosphate followed by vascularisation and finally by resorption which coincides with significant clinical pain. The post calcific stage is demonstrated by the collagenisation of the lesion by fibroblasts [26]. Intra-operatively, the gross specimens of CT can be either in the form of a sandy tough mass or a toothpaste-like fluid or an amorphous mass composed of many small round or ovoid bodies [27]. The material of these deposits has been identified to be calcium carbonate apatite [28]. This carbonate apatite has been further classified as an A and B-type apatite [29]. Chiou et al. [30] studied the chemical components in CT and found that both types of the carbonate apatite varied in quantities during the formative, resting and resorption phases. Histochemical studies have demonstrated the presence of extracellular matrix vesicles near calcified deposition of the RC [26, 31, 32] and the authors have tried to correlate this finding in the pathogenesis of CT. Normally, the vesicles are inhibited from mineralization but in the presence of any pathology, the inhibitory stimulus may be lost leading to vesicles getting mineralized.

Radiographically, these deposits have been classified by different authors as described in Table 1.

**Table 1:** Radiological classification of the calcific tendinitis of the shoulder according to the current literature evidence

References	Radiographic criteria	Classification
Bosworth et al. [7]	Size	Large (>1.5 cm)
		Medium (in between)
		Small (rarely seen)
Depalma et al. [3]	Morphologic features	Type I (fluffy, amorphous and ill defined)
		Type II (defined and homogeneous)
Gartner et al. [33, 34]	Morphologic features	Type I (well demarcated, dense)
		Type II (soft contour and dense or sharp contours and transparent)
		Type III (soft contours, translucent and cloudy)
Mole’ et al. [35]	Morphologic features	Type A (dense, rounded, sharply delineated)
		Type B (multilobular, radiodense, sharp)
		Type C (radiolucent, heterogeneous, irregular outline)
		Type D (dystrophic calcific deposits)

Maier M et al. [36] assessed the intra- and interobserver reliability of the various classification systems using plain radiographs and CT scans and concluded that all the scores showed insufficient reliability and reproducibility. Although marginal improvement could be seen using CT scans it still remained statistically insignificant to be recommended as a routine investigation.

## Diagnostic imaging

The first imaging modalities to identify CT were X-ray and ultrasound, as calcium deposits are readily identifiable on both. Radiograms should be performed in anterior-posterior (AP)—neutral, internal rotation and external rotation—axillary and outlet view. On radiographs calcific deposits appear homogeneous, amorphous densities without trabeculation, which allows a differentiation from heterotopic ossification or accessory ossicles [37]. Most of calcifications are ovoid, and the margins may be smooth or ill-defined. Ultrasound (US) is advantageous in the diagnosis of CT as it helps to detect other associated conditions as well such as rotator cuff tears and long head of the biceps (LHB) pathologies [38]; moreover, it also characterizes deposit consistency, their tendon location, and can be helpful to assist injections and bursal lavage [39]. According to the morphology of the calcium deposit, US has been used to classify the different type of CT due to its ability to discriminate between well defined calcifications with strong shadowing, and those with faint or absent shadowing. Chiou et al. [40] classifies calcific depositions into four shapes: an arc shape (echogenic arc with clear shadowing), a fragmented or punctate shape (at least two separate echogenic spots or plaques, with or without shadowing), a nodular shape (echogenic nodule without shadowing), and a cystic shape (a bold echogenic wall with an anechoic area, weak internal echoes or layering content). Conditions associated with non arc-shape calcifications include hypervascularity, widening of subacromial-subdeltoid bursa and the large size of calcifications. High resolution US in combination with color Doppler can differentiate between formative or resorptive status. In the resorptive phase, the deposits are nearly liquid and can be successfully aspirated. US has been also used with success in overhead athletes to identify CT showing a prevalence greater than that reported in the general population and that the presence of calcific tendinopathy correlates positively with age [41]. CT scan and MRI should be reserved for doubtful cases [42]. Computed tomography has an excellent resolution to detect calcium deposit as high density foci of solid stippled or amorphous character, but the cost and the exposure to radiation limit its use. MRI should not be used as a first line imaging modality, because deposits appear as vague regions of low signal on T1 and T2, and can be missed. Some enhancement around the deposit can be seen after contrast, and surrounding areas of hyperintensity on T2, due to peripheral edema or subacromial-subdeltoid bursal fluid are possible. MRI is advisable when the deposit is so large as to produce a strong shadow on US thus confusing it with RCTs.

## Complications

### Pain

The reason why pain has been considered as a complication in this review is due to the fact that this condition remains primarily asymptomatic in most of the patients [6]. When CT becomes symptomatic, the pain is extremely severe and is typically shooting type in the area of the shoulder with no radiation to elbow or hand [43]. In the acute phase, the pain tends to be so severe so as to allow only limited shoulder motion with marked tenderness. In the chronic or subacute phase, pain can be severe but generally shoulder motion is allowed [44]. The cause of occurrence of pain in CT is either due to an inflammatory response to the local chemical pathology or to direct mechanical irritation [45]. Neer classically described four types of pain peculiar to calcium deposition. First is the pain that is caused by the chemical irritation of the tissue by calcium. The second is the pain caused by tissue pressure due to its swelling. The third is an impingement-like pain caused by bursal thickening or irritation by the deposit itself. The fourth is the pain caused by a chronic stiffening of the glenohumeral joint due to voluntary prolonged immobilization by the patient to avoid possible irritation by the deposits with abduction or overhead activities [46]. Substance P is involved in the pain transmission caused by the stimulation of A delta/C fibers by certain noxious stimuli in the dorsal horn of the spinal cord. It is also contained in the small sensory neurons of the peripheral tissue. It’s release from the sensory neurons play a significant role in mediating neurogenic inflammation [47]. Gotoh M et al. [47] studied the relation of the amount of substance P in the subacromial bursa and the shoulder pain in patients with rotator cuff diseases with radioimunoassay and immunohistochemistry. He found an increase in the number of immunoreactive nerve fibres in the synovial tissue of patients with rotator cuff diseases. These fibres were predominantly located around the blood vessels, suggesting an active role in its regulation and subsequent inflammation. He also hypothesized that certain mechanical (impingement) and chemical (bursitis) factors could be a source for the noxious stimuli inducing increased amounts of substance P in the afferent nerves. The conclusion of his study was that the subacromial bursa was the site associated with shoulder pain caused by rotator cuff disease.

We suggest to pay special attention to patients with persistent pain due to chronic CT. This subpopulation requires periodical outpatient visit (every 4 months) to exclude stiffness and monitor the evolution of calcium deposit with ultrasound; in addition, radiograms should be performed annually to assess the morphology of the deposit and its relationship with the underneath bone. NSAIDs are recommended when the pain score is more than 5 on a Visual Analogic scale (0–10). A standard program of physiotherapy including self aided mobilization and home exercises are prescribed to prevent stiffness. ESWT may be advised to foster calcium resorption, while other physical therapies (Laser, Transcutaneous electrical nerve stimulation) may help to treat associated LHB tendinopathies.

In addition, we do believe that some of the other complications listed below could be an important source of chronic and resistant pain in CT.

### Adhesive capsulitis

**Fig. 2:**
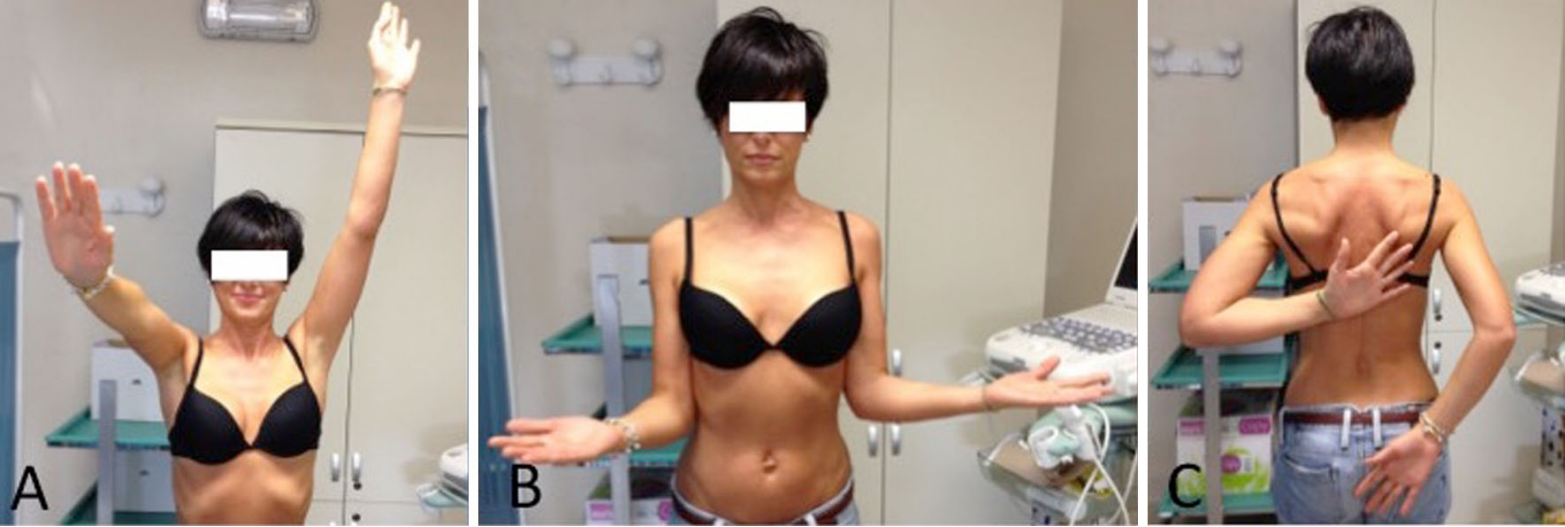
**a**–**c** Active range of motion in a young lady with chronic calcifying tendinitis of the SS. At 2 months from the onset of pain she developed a stiff shoulder that required 6 months of manual physiotherapy for full recovery of shoulder motion

Although the etiology of adhesive capsulitis is still not well understood, the pathophysiology has been much better explained over the years [48]. Two forms are commonly described: primary and secondary forms. While immobility is an important factor in the etiology, some case series have shown no predisposing factors for the primary form [49–52]. The secondary form is the more common type and can be precipitated by extrinsic factors or systemic diseases [53–58] or from intrinsic diseases in which CT is an important cause [59, 60]. Despite the efforts in elucidating this condition, there is still difficulty in deciding if the capsule abnormalities have resulted from inflammation of the surrounding structures or vice versa [48]. The amorphous calcium deposits lead to pain and dysfunction in the shoulder. The physical characteristics of these deposits influence the clinical presentation of the patient. If the calcium is in liquid state, an acute process is generally manifested with severe pain being the most important symptom. But if the deposit is dry and hard, a chronic form is usually seen in which the pain is superseded by a limited range of shoulder motion with a secondary frozen shoulder being the most important sequela (Fig. 2a–c) [61]. Shoulder stiffness is not well tolerated by patients with CT and must be treated with standard manual therapies to gain a complete recovery of shoulder mobility. Shoulder stiffness associated with CT is not easy to resolve and may require long-term rehabilitation, NSAIDs consumption and articular steroid injections in resistant cases. Therefore, we recommend to each physician who deals with cases of CT to precociously recognize any case of stiffness and address it appropriately.

Another interesting association of stiffness and CT is found in the post operative phase in arthroscopy. In a study by Jacobs et al. [62] the incidence of frozen shoulder after surgery was 18 % and the cause was considered to be the irritation of the glenohumeral capsule by residual calcium debris and hence thorough lavage was recommended to avoid such a possibility. Although he did not have literature evidence to support his claim, this assumption may not be entirely misplaced. In the section on pain previously described, one cause for it was considered to be stiffening due to voluntary prolonged immobilization. Conversely, the pain produced could further limit the compliance of the patient with respect to physiotherapy and rehabilitation thus producing a vicious cycle. Overall this association would usually lead to a prolonged recovery phase with regards to strength and motion.

### Rotator cuff tears

**Fig. 3:**
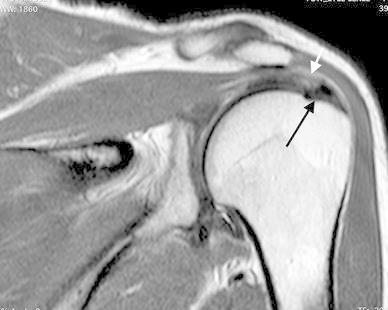
T1-weighted coronal oblique MRI shows a solid calcium deposit at the insertion of the SS (*black arrow*) with partial tear of the related tendon on the bursal side (*white arrow*)

This pathology can coexist either pre-operatively or intra-operatively. In the pre-operative setting, in the earlier times it was strongly believed that there could not be a coexistence of both the entities [63] but with time this theory became disputed. Kernwein showed with arthrography a 90 % probability to reveal a rotator cuff tears (RCTs) in a patient older than 40 years with CT. He explained that large calcium deposits can rupture thus leading to complete RCTs [64]. Wolfgang reported an incidence of 23 % of CT in his subjects who underwent surgical repairs of RC tear [65]. Hsu also studied the relationship between these 2 pathologies and finally summarized his findings into 12 observations. His study showed a 28 % probability of coexistence of CT and RCTs. He observed the tears to be associated with smaller sized deposits and that the integrity of the cuff, the tear pattern, the shape, site and sex were significantly related to the texture of the calcific deposit [66]. Progression from calcifying tendinitis to RCTs has been also reported by Gotoh et al. [67]. On the basis of these research findings we may speculate that inflammation following a cuff tear can lead to resolution of the calcium deposits and hence may produce a radiographic picture of a small sized deposit (Fig. 3). However, there is no literature evidence to support this belief.

**Fig. 4:**
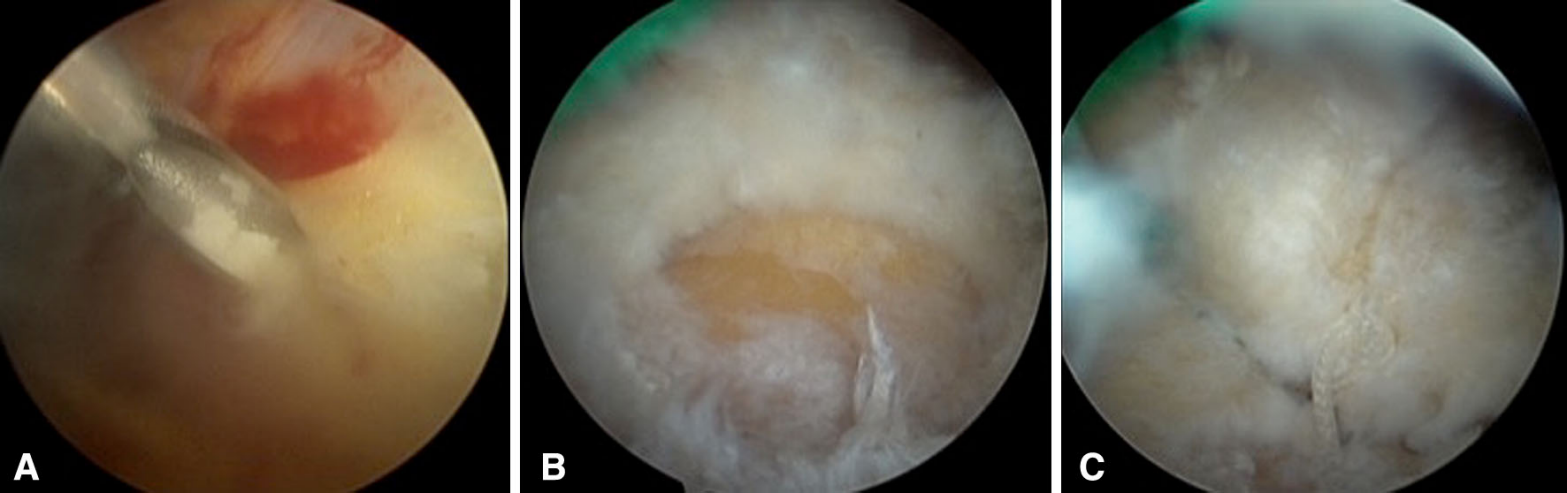
Arthroscopic steps in a patient with chronic calcific deposit of the SS tendon. **a** Intraoperative needling to identify the site of deposit and delimit the amount of tendon to be removed, **b** full thickness insertional SS tear produced after complete removal of calcium deposit, **c** SS reattached on its footprint using a suture anchor (Cross FT 4.5 mm, Linvatec, Largo, FL—USA)

The second association of RC tears with CT is in the intra-operative findings. Usually, removal of the calcium deposits leaves various degrees of RC defects which depend on the amount of the deposit present and the extent of resection. If the defects are full thickness or large partial thickness then intra-operative repair is recommended (Fig. 4). There is no general consensus in the current literature regarding the extent of the resection of the deposits to be done. Some authors have suggested complete removal of the deposits with repair of the rotator cuff if necessary as it is believed that there is an inverse relation between clinical outcomes and any residual calcium deposits [22, 68–70]. In contrast, other researchers have reported good clinical outcomes with minimal tendon damage [1, 24, 62, 71, 72]. These studies were based on the hypothesis that the pain in CT is due to edema and increased intratendinous pressure as a result of calcification and thus just tendon decompression would suffice. Also, the same authors asserted that most of the patients with remnant deposits tended to show progressive resorption over time. Balke M et al. [1] in a mid term follow up study (2–13 years) reported worse clinical outcomes in the operated cases of CT, who also showed a high rate of partial supraspinatus tears. Nevertheless this study was the object of criticism for the involvement of multiple surgeons and lack of account for residual calcifications in the follow up [73]. Seil R et al. [72] in a follow up of over 24 months found complete resolution of residual calcium in all his cases except 2 along with an excellent clinical score in more than 90 % of the patients. Conversely, Porcellini et al. [22] in a follow up of over 36 months found that the Constant score was significantly lesser in those patients with persistent calcium deposits. Yoo et al. [69] noticed significant pain relief in 30 out of 35 patients at 6 months after surgery which was considered to be due to aggressive surgical debridement; furthermore it was interesting to note that the residual calcium deposits in 6 patients showed complete resolution with time.

### Greater tuberosity osteolysis

**Fig. 5:**
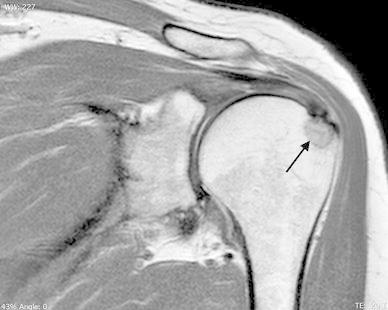
T1-weighted coronal oblique MRI highlights a greater tuberosity osteolysis (*black arrow*) in a case with a calcium deposit of the SS in contact with the bone

This is an extremely uncommon complication of CT. Sometimes, the classical course of CT may be altered leading to a longer duration of symptoms and greater functional impairment [74]. Osteolytic lesions (OL) of the tuberosities can be one of such causes [22, 42, 75]. Flemming G et al. [42] described a diffuse form of heterogeneous calcification, deep within the tendon near its insertion as a reason for the worst and most persistent symptoms. Seil R et al. [72] tried to correlate the persistent pain experienced by some patients to the penetration of calcium into bone as a result of the cortical erosion and the biochemical effects of bone lysis. Porcellini G et al. [75] studied a large series of such patients. MRI was used as the imaging modality of choice for detection of osteolysis as it was shown to be more reliable in demonstrating contact between the deposit and the bone (Fig. 5). He found that those calcium deposits which were in contact with the tuberosities consistently produced cortical lesions. These lesions were not related to the shape and size of the deposits or to the sex, age and occupation of the patients. Also, he found a significant correlation between clinical and imaging findings i.e. the more severe the osteolytic lesions, especially those extending to the lateral facet, the less improvement noticed at the final follow-up. Finally, he concluded that this subset of patients had less favorable outcomes with respect to the degree and time of functional recovery. Overall, in presence of OL the prognosis of patients with CT is worse and may be particularly resistant to the common conservative therapies. Although this subset of patients gain lower postoperative clinical scores, surgical approach should be considered in case of severe pain when all the other non-operative treatment fails; arthroscopic approach allow to identify the site of OL and to perform an accurate cleaning of the bone that is useful to reduce pain and improve shoulder function.

### Ossifying tendinitis

**Fig. 6:**
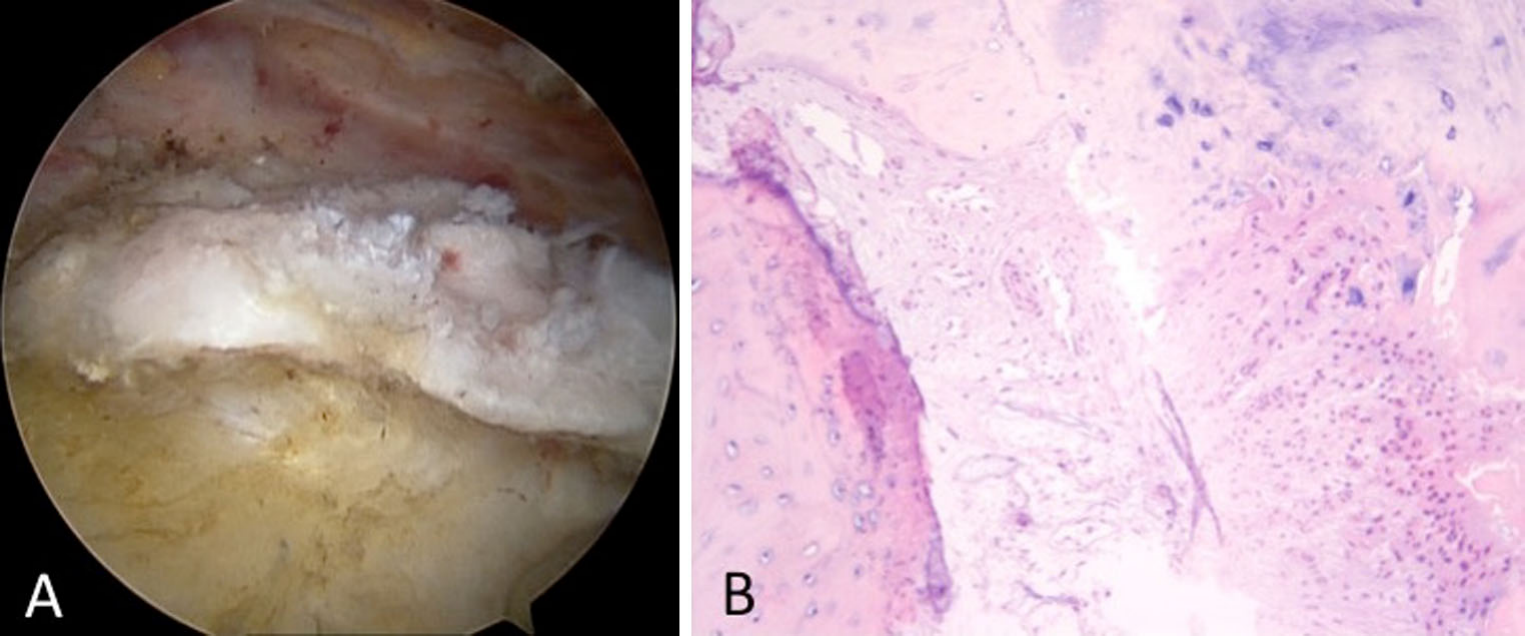
**a** Arthroscopic finding of recurrence of calcific tendinitis of the rotator cuff in the form of ossifying tendinitis, **b** histologic examination confirmed the diagnosis showing tendinous tissue mixed with areas of chondroid and bone metaplasia

This is an extremely rare complication of CT and to date only one article has been found to be published in a broad based literature search [6]. This is a type of heterotopic ossification characterized by deposition of hydroxyapatite crystals in a histologic pattern of mature lamellar bone [76]. It is usually associated with surgical intervention or trauma with the Achilles tendon, distal biceps and in gluteus maximus tendons. Merolla G et al. [6] studied two such cases in shoulder who had an arthroscopic removal of CT and subsequently was histologically proved to be ossifying tendinitis (OT) (Fig. 6a, b). Incidentally, both the cases had an initial arthroscopic removal of a routine CT with subsequent recurrence which manifested itself as ossifying tendinitis. He hypothesized that the ossifications found could have been the result of a transformation of mesenchymal cells to bone-forming cells in response to the surgical excision of calcium deposit and suturing of the tendon during the index arthroscopic procedure. He recommended to consider arthroscopic excision of calcium deposits with caution and to be meticulous during the subacromial debridement of calcific foci to minimize the risk of recurrence. OT is a very rare complication of CT but the actual rate is unknown because of the very few patients have who undergone arthroscopic second-look in presence of radiographic evidence of recurrence of CT. We do believe that the number of cases with this complication is underestimated and we advise to be cautious in dealing with such cases and to refer the doubtful cases with persistent pain for more than a year to the surgeon.

## Conclusions

The ideal treatment for the CT of the shoulder is not well established and for some aspects still controversial. The clinical course may be complicated by several conditions that should be diagnosed and treated when we manage a patient with CT of the RC. Whereas pain and stiffness are generally recognized and treated, the risk of RC tears ìs not well considered and the related surgical approach is a concern. Greater tuberosity osteolysis is less known and often not identified on radiograms or ultrasound, therefore, we would suggest to investigate with MRI in those patients with persistent chronic pain and doubtful standard X-ray. Finally, ossifying tendinitis is very rare and only recently reported as complication of CT that should be considered and investigated with X-ray in subjects with CT already treated with conservative or operative measures. We do believe that this review gives a quick summary of the potential complications of the CT, inviting all professionals (orthopaedic surgeons, physiatrists, radiologists and physiotherapists) who deal with this disease to consider not only the regular course of the CT but also the complications that must be identified and treated as well as possible.
